# Active *Helicobacter pylori* Infection and Survival Outcomes in De Novo Metastatic Colorectal Cancer: A Retrospective Cohort Study

**DOI:** 10.1111/hel.70150

**Published:** 2026-07-06

**Authors:** Sait Kitapli, Ali Alkan, Ozgur Tanriverdi

**Affiliations:** ^1^ Medical Oncology Clinic Mugla Education and Research Hospital Mugla Turkey; ^2^ Department of Medical Oncology Mugla Sitki Kocman University Faculty of Medicine Mugla Turkey

**Keywords:** biomarker, Cox regression, *Helicobacter pylori*, metastatic colorectal cancer, neutrophil‐to‐lymphocyte ratio, overall survival, prognosis, survival analysis, systemic inflammation, tumor sidedness

## Abstract

**Background:**

The prognostic significance of *Helicobacter pylori* ( *H. pylori* ) infection in metastatic colorectal cancer (mCRC) remains uncertain. Most prior studies have focused on colorectal neoplasia risk and have relied on serological markers rather than assessment of active infection. We evaluated the association between histologically confirmed *H. pylori* status and clinicopathological features, systemic inflammatory markers, and survival outcomes in patients with de novo mCRC.

**Methods:**

In this retrospective single‐center cohort study, consecutive patients diagnosed with de novo mCRC between September 2011 and December 2025 who underwent synchronous upper gastrointestinal endoscopy at diagnosis were included. *H. pylori* status was determined histopathologically. Overall survival (OS) was estimated using the Kaplan–Meier method and compared with the log‐rank test. Prognostic factors were analyzed using univariable and multivariable Cox proportional hazards models with prespecified sensitivity and exploratory interaction analyses.

**Results:**

Among 168 patients, 77 (46%) were *H. pylori* positive. Right‐sided tumors were more frequent in *H. pylori* –positive patients (43% vs. 17%, *p* < 0.001), as was BRAF mutation (12% vs. 3%, *p* = 0.044). Baseline neutrophil‐to‐lymphocyte ratio was higher in the *H. pylori* –positive group (median 4.34 vs. 2.74, *p* = 0.002). Median OS was longer in *H. pylori* –positive patients (33 vs. 19 months; log‐rank *p* < 0.001). In multivariable analysis, right‐sided tumor localization (HR 2.11, 95% CI: 1.648–2.716; *p* < 0.001), RAS mutation (HR 1.94, 95% CI: 1.612–2.794; *p* < 0.001), and *H. pylori* negativity (HR 1.94, 95% CI: 1.569–2.812; *p* < 0.001) remained statistically significant. These findings were consistent across sensitivity analyses. Significant interactions were observed for tumor sidedness (*p* < 0.001) and NLR category (*p* = 0.002).

**Conclusions:**

Histologically confirmed *H. pylori* positivity was associated with a distinct clinicopathological profile and longer overall survival in patients with de novo mCRC. Given the observational design, these findings should be interpreted as hypothesis‐generating and require validation in prospective and multi‐center studies.

## 1 Introduction

Colorectal cancer (CRC) remains a major global health challenge and continues to rank among the leading causes of cancer‐related mortality worldwide. Its pathogenesis is multifactorial, reflecting a complex interplay between host genetic susceptibility, environmental exposures, systemic immune responses, and the dynamic composition of the intestinal microbiome [1]. While traditional research has largely focused on established genetic and lifestyle‐related risk factors, increasing attention has been directed toward the potential role of specific microbial agents in modulating the tumor microenvironment and influencing the clinical course of metastatic colorectal cancer (mCRC). This evolving field is of growing translational and prognostic relevance [2, 3, 4].

*Helicobacter pylori* ( *H. pylori* ) is unequivocally classified as a class I carcinogen in gastric malignancies. However, its impact beyond the stomach, particularly in colorectal carcinogenesis and disease progression, remains incompletely understood [5]. Several studies have reported an association between *H. pylori* seropositivity and colorectal adenomas or early‐stage neoplasia, whereas data regarding its role in advanced and metastatic disease are limited and, in some cases, conflicting. These observations suggest that the biological consequences of *H. pylori* infection may vary according to disease stage and tumor–host interactions, and therefore findings derived from early carcinogenesis models should not be directly extrapolated to established metastatic disease [6, 7, 8, 9]. But it remains unclear whether *H. pylori* primarily represents a distant inflammatory stimulus or whether it may act as a systemic immune modulator capable of influencing tumor behavior.

A potential biological rationale for a role of *H. pylori* in CRC progression may be related to its capacity to induce chronic, low‐grade systemic inflammation and to modulate host immune responses. Experimental and translational studies have shown that *H. pylori* infection can influence T‐cell polarization and activate pro‐inflammatory signaling pathways, including IL‐6/STAT3 and NF‐κB, which are known to be involved in colorectal tumor biology [10, 11]. Beyond these pro‐tumorigenic mechanisms described in early carcinogenesis, chronic microbial exposure has also been proposed to affect immune surveillance and the tumor–immune equilibrium in advanced disease. Interestingly, emerging hypotheses suggest that persistent immune activation associated with *H. pylori* may, in certain biological contexts, enhance antitumor immune surveillance or alter the gut–microbiome axis in a manner that could influence survival outcomes in selected patient subsets [12, 13, 14].

mCRC is characterized by marked molecular heterogeneity. Alterations in RAS and BRAF genes, HER2 amplification/overexpression, and deficiencies in DNA mismatch repair (MMR) pathways not only define distinct clinical phenotypes but also have important predictive and prognostic implications [15]. Despite the established relevance of these biomarkers, data evaluating their potential interaction with *H. pylori* infection are lacking [16]. Similarly, the relationship between *H. pylori* status and systemic inflammatory markers, such as the neutrophil‐to‐lymphocyte ratio (NLR) and C‐reactive protein (CRP), has not been adequately explored in real‐world clinical cohorts [17, 18].

The present study was designed to address these gaps in the current literature. Taking advantage of a unique cohort of patients with mCRC who underwent synchronous gastroscopic evaluation at the time of diagnosis, we aimed to investigate the potential associations between *H. pylori* status and primary tumor localization, molecular characteristics, and systemic inflammatory markers. Most importantly, we sought to evaluate the independent prognostic value of *H. pylori* infection on overall survival, thereby providing a clinically oriented perspective on the interaction between chronic microbial exposure and tumor biology in metastatic colorectal cancer.

## 2 Methods

### 2.1 Study Design and Patient Selection

This retrospective, single‐center cohort study was conducted at the Medical Oncology Department of Muğla Training and Research Hospital. The study commenced after ethical approval was obtained from the Muğla Sıtkı Koçman University Faculty of Medicine and Health Sciences Ethics Committee on January 16, 2026, with approval number 250115/82. The study was approved by the institutional ethics committee and conducted in accordance with the Declaration of Helsinki.

Consecutive patients diagnosed with mCRC between September 2011 and December 2025 were screened through the institutional electronic database.

Inclusion criteria were histologically confirmed colorectal adenocarcinoma, presence of distant metastasis at initial diagnosis, age ≥ 18 years, and availability of synchronous upper gastrointestinal endoscopy with histopathological evaluation for *H. pylori* . At our institution, upper gastrointestinal endoscopy is frequently performed at the time of diagnosis in patients with newly diagnosed metastatic CRC, particularly in the presence of anemia, upper gastrointestinal symptoms (e.g., dyspepsia, weight loss), or as part of baseline nutritional and bleeding risk assessment prior to systemic therapy initiation. Therefore, although not universally applied to all patients, endoscopy represents a common component of baseline evaluation in real‐world clinical practice.

Given that endoscopic evaluation was not performed systematically in all patients, the potential for selection bias related to endoscopy availability was considered.

Patients whose diagnosis was established following emergency surgery, those who underwent metastasectomy or local ablative treatment at baseline, and those without available histopathological data for *H. pylori* status were excluded.

**FIGURE 1:**
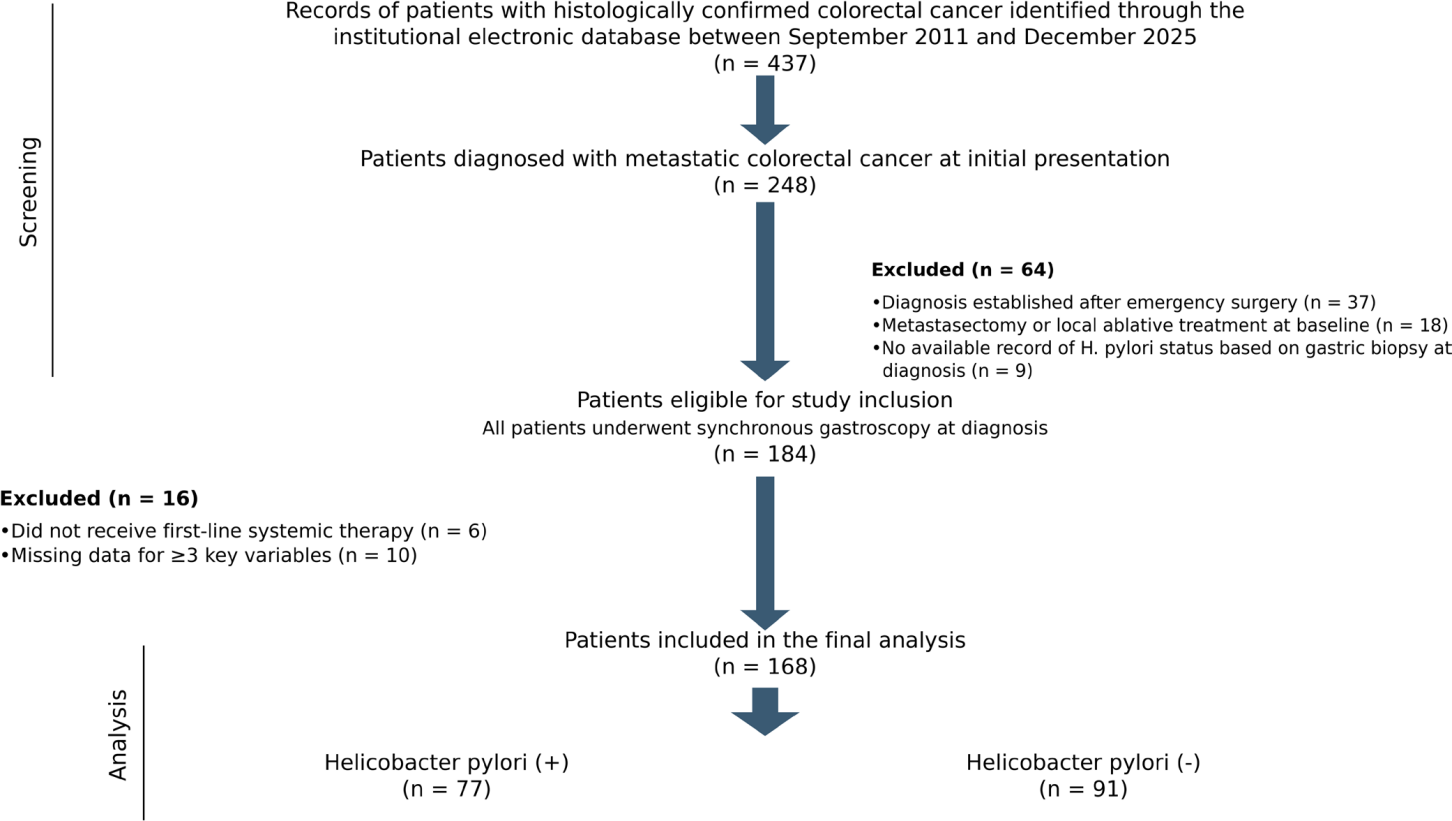
Study flow diagram. Flow chart illustrating the selection of patients with metastatic colorectal cancer from the institutional database. Among 437 patients with histologically confirmed colorectal cancer, 248 were diagnosed with metastatic disease at initial presentation. After exclusion of patients diagnosed after emergency surgery (*n* = 37), those who underwent metastasectomy or local ablative treatment at baseline (*n* = 18), and those without available histopathological *Helicobacter pylori* status (*n* = 9), 184 patients were eligible. A further 16 patients were excluded due to lack of first‐line systemic therapy (*n* = 6) or missing data for ≥ 3 key variables (*n* = 10). The final study population consisted of 168 patients, including 77 *Helicobacter pylori*–positive and 91 *Helicobacter pylori*–negative cases.

Among the eligible population, patients who did not receive first‐line systemic therapy or had missing data for ≥ 3 key variables were further excluded from survival analyses. A total of 168 patients met the study criteria and were included in the final analysis (Figure 1). To assess potential selection bias, baseline characteristics of patients included in the final analysis were compared with those of patients who were excluded due to lack of endoscopic evaluation or missing *H. pylori* status (Table S1). Because histological assessment of *H. pylori* required gastric biopsy obtained during upper gastrointestinal endoscopy, a separate non‐endoscoped comparison group could not be constructed using the same exposure definition. Because synchronous upper GI endoscopy was performed according to clinical indications rather than systematically, the study cohort should be regarded as a selected subgroup of patients with de novo metastatic CRC. Comparisons between included and excluded patients were performed to characterize potential differences in cohort composition; however, these analyses cannot eliminate residual selection bias or establish representativeness of the study population.

### 2.2 Data Collection and Clinical Parameters

Demographic, clinical, and treatment‐related data were extracted from electronic medical records. Variables included age, sex, smoking status (never, former, or active smoker), comorbidities, and chronic proton pump inhibitor (PPI) use. Information on PPI use was recorded due to its potential impact on *H. pylori* detection.

Primary tumor localization was categorized as right‐sided colon (cecum to transverse colon), left‐sided colon (splenic flexure to sigmoid colon), and rectum. Metastatic involvement (liver, lung, bone, brain, distant lymph node, and adrenal gland) was determined based on baseline computed tomography (CT) or positron emission tomography (PET/CT) imaging. Patients could have more than one metastatic site at diagnosis. Tumor burden was additionally characterized by the number of metastatic sites (1, 2, or ≥ 3), as recorded at baseline imaging.

Because all patients had de novo metastatic disease at presentation, prior adjuvant therapy was not applicable in this cohort. Details of first‐line systemic treatment, including chemotherapy backbone, biologic agent, maintenance strategy, dose reductions, treatment cessation, and grade 3–4 toxicity, were recorded. Detailed data on first‐line systemic treatment exposure were collected because treatment initiation and early disease control represent the major determinants of OS in de novo metastatic CRC. First‐line chemotherapy consisted predominantly of oxaliplatin‐based regimens (mFOLFOX6 or CAPOX). A small minority of patients received irinotecan‐based therapy (FOLFIRI) or single agent (capecitabine or irinotecan), mainly due to pre‐existing clinical conditions such as suspected oxaliplatin intolerance (e.g., neuropathy). Second‐line treatments were also recorded as a study variable.

For categorical comparisons, percentages were calculated based on available data, and patients with missing values were excluded from the corresponding analyses.

### 2.3 Histopathological and Molecular Analysis

*H. pylori* status was determined by histopathological examination of gastric biopsy specimens obtained during synchronous endoscopy. Gastric biopsies were obtained according to institutional practice, typically including at least two samples from the antrum and two from the corpus. When available, biopsy sampling followed principles consistent with the updated Sydney system. All specimens were evaluated by experienced gastrointestinal pathologists using standard staining methods (Giemsa or Warthin–Starry) as part of routine diagnostic assessment at the time of cancer diagnosis, and pathologists were not aware of patient survival outcomes at the time of *H. pylori* assessment.

Chronic PPI use was specifically recorded and incorporated into clinicopathological comparisons and interaction analyses. Review of available endoscopy records showed that, in most patients, no gastroscopy other than the index examination performed at colorectal cancer diagnosis had been recorded in the preceding 2 years within the institutional system. Structured medication data focused on chronic treatments; short‐term antibiotic, bismuth, and eradication‐treatment exposure was variably available in the electronic record and could not be analyzed separately.

Molecular analyses were performed as part of routine clinical practice. RAS (KRAS and NRAS) and BRAF mutation status were assessed using either next‐generation sequencing or PCR‐based assays according to tissue availability. MMR status was evaluated by immunohistochemistry and/or PCR‐based microsatellite instability testing. HER2 amplification/overexpression status was initially assessed by immunohistochemistry, and equivocal (2+) cases were confirmed by silver in situ hybridization.

### 2.4 Laboratory Measurements

Baseline laboratory parameters were obtained within 14 days prior to the initiation of any systemic therapy, under clinically stable conditions and in the absence of laboratory findings suggestive of acute metabolic derangement, active infection, or other concomitant inflammatory conditions.

Hematological variables, including hemoglobin (g/dL; reference range: 12–16 for females and 13–17 for males), total leukocyte count (×10^3^/μL; 4000–10,000), absolute neutrophil count (×10^3^/μL; 1500–7500), absolute lymphocyte count (×10^3^/μL; 1000–3000), and platelet count (×10^3^/μL; 150,000–400,000), were analyzed using an automated hematology analyzer (Sysmex XN‐1000, Sysmex Corporation, Kobe, Japan). Biochemical parameters, including serum albumin (g/dL; 3.5–5.0), lactate dehydrogenase (LDH, U/L; 135–225), CRP (mg/L; < 5), and uric acid (mg/dL; 3.4–7.0), were measured on a Cobas 8000 modular analyzer (Roche Diagnostics, Mannheim, Germany).

The NLR was calculated as the ratio of the absolute neutrophil count to the absolute lymphocyte count. For subgroup and regression analyses, NLR was categorized using a predefined cut‐off value of ≥ 3 based on prior literature.

### 2.5 Outcome Definition

The primary endpoint was overall survival (OS), defined as the time from the date of metastatic diagnosis to death from any cause or last follow‐up. The time from the start of first‐line treatment to progression was defined as progression‐free survival (PFS) and constituted the secondary endpoint of the study. Progression for PFS was determined retrospectively from cross‐sectional imaging reports and treating‐physician documentation in the electronic medical record. Follow‐up imaging was performed according to routine clinical practice, generally at 8‐ to 12‐week intervals or earlier when clinically indicated. Death without documented radiologic progression was counted as a PFS event. Formal RECIST‐based reassessment was not performed, and progression status was derived from routine radiologic reports and treating‐physician documentation.

### 2.6 Statistical Analysis

Continuous variables were expressed as median with interquartile range (IQR) and compared between groups using the Mann–Whitney *U* test, as the distribution of the variables was not assumed to be normal. Categorical variables were summarized as frequencies and percentages and compared using the Chi‐squared test or Fisher's exact test, as appropriate. OS was estimated using the Kaplan–Meier method and differences between groups were assessed with the log‐rank test. Subgroup survival analyses according to *H. pylori* status were performed using the same approach. Patients with missing or unknown values were excluded from the corresponding subgroup analyses and were not treated as a separate category. For variables with more than two categories, pairwise comparisons were performed with Bonferroni correction after exclusion of missing or unknown values.

Univariable Cox proportional hazards regression analysis was conducted to identify potential prognostic factors for OS. Covariates included in the multivariable model were prespecified on the basis of clinical relevance and prior evidence and were entered simultaneously. Cox models were fitted on a complete‐case basis among patients included in the final analysis. The proportional hazards assumption was evaluated using log–log survival plots and was not violated. The proportional hazards assumption was assessed for each covariate. Formal interaction analyses were performed within the Cox proportional hazards model by including multiplicative interaction terms between *H. pylori* status and the relevant clinicopathological variables. Given the modest sample size and limited numbers within some clinically relevant subgroups, these interaction analyses were considered exploratory and hypothesis‐generating. The statistical significance of the interaction was assessed using the Wald test. Treatment‐related confounding was addressed by including targeted treatment and chemotherapy backbone in the primary multivariable model and by performing prespecified sensitivity analyses incorporating metastatic burden, NLR, an oxaliplatin‐based restricted cohort in which chemotherapy regimen, and second‐line treatment was additionally modeled.

To assess the robustness of the primary model, prespecified sensitivity analyses were performed using alternative multivariable models in which liver metastasis was replaced by the number of metastatic sites and the NLR was removed to explore its potential role as a mediator or confounder in the association between *H. pylori* status and OS. An additional sensitivity analysis was performed by restricting the cohort to patients receiving oxaliplatin‐based chemotherapy.

All statistical tests were two‐sided and a *p* value < 0.05 was considered statistically significant. Statistical analyses were performed using SPSS software (version 25.0; IBM Corp., Armonk, NY, USA).

## 3 Results

### 3.1 Patient Characteristics and Clinical Profile

A total of 168 patients with mCRC were included in the final analysis. *H. pylori* positivity was detected in 77 patients (46%), while 91 patients (54%) were negative. Baseline clinicopathological characteristics according to *H. pylori* status are summarized in Table 1.

**TABLE 1:** Comparison of clinicopathological characteristics according to *Helicobacter pylori* status.

Characteristics	*H. pylori* positive (*n* = 77)	*H. pylori* negative (*n* = 91)	*p*
Gender			
Female	30 (39)	28 (31)	0.329
Male	47 (61)	63 (69)	
Age			
< 65	34 (44)	41 (45)	0.379
65 and over	43 (56)	50 (55)	
Smoking habits			
Never/Ex‐smoker	58 (75)	66 (72)	0.406
Active smoker	19 (25)	25 (28)	
Comorbidity			
Present	45 (58)	48 (53)	0.724
Absent	29 (38)	38 (42)	
Unknown	3 (4)	5 (6)	
Proton pump inhibitors use before diagnosis			
Present	30 (39)	36 (40)	0.273
Absent	41 (53)	41 (45)	
Unknown	6 (8)	14 (15)	
Family history of cancer			
Present	10 (13)	15 (17)	0.532
Absent	24 (31)	33 (36)	
Unknown	43 (56)	43 (47)	
Performance status (ECOG)			
0	8 (10)	12 (13)	0.396
1	67 (87)	76 (83)	
2	2 (3)	3 (4)	
Primary tumor localization			
Right colon	33 (43)	16 (17)	**< 0.001**
Left colon	35 (45)	57 (63)	
Rectum	9 (12)	18 (20)	
Chemotherapy backbone (first‐line)			
mFOLFOX6	37 (48)	47 (52)	0.637
CAPOX	32 (42)	37 (41)	
FOLFIRI	3 (4)	3 (3)	
Other (single‐agent^a^)	5 (6)	4 (4)	
Chemotherapy subgroup (first‐line)			
Oxaliplatin‐based	69 (90)	84 (92)	0.548
Irinotecan‐based	6 (8)	5 (6)	
Capecitabine	2 (2)	2 (3)	
Targeted treatment (first‐line)			
Anti‐VEGF	48 (62)	46 (51)	0.114
Anti‐EGFR	29 (38)	45 (49)	
Number of metastatic sites			
1	13 (17)	20 (22)	0.096
2	27 (35)	30 (33)	
3 and over	37 (48)	41 (45)	
Liver metastasis status			
Present	46 (60)	59 (65)	0.301
Absent	31 (40)	32 (35)	
Lung metastasis status			
Present	33 (43)	32 (35)	0.342
Absent	44 (57)	59 (65)	
Bone metastasis status			
Present	11 (14)	11 (12)	0.819
Absent	66 (86)	80 (88)	
Brain metastasis status			
Present	1 (1)	6 (7)	0.183
Absent	76 (99)	85 (93)	
Distant lymph node metastasis status			
Present	24 (31)	37 (41)	0.270
Absent	53 (69)	54 (59)	
Adrenal gland metastasis status			
Present	17 (22)	20 (22)	0.269
Absent	60 (78)	71 (78)	
RAS mutation status			
Mutant	25 (33)	29 (32)	0.532
Wild	52 (67)	62 (68)	
BRAF mutation status			
Mutant	9 (12)	3 (3)	**0.044**
Wild	68 (88)	88 (97)	
Microsatellite status			
Stable	62 (80)	66 (73)	0.472
Instable	4 (6)	6 (7)	
Unknown	11 (14)	19 (21)	
HER2 amplification/overexpression			
Present	1 (1)	1 (1)	0.312
Absent	43 (56)	27 (30)	
Unknown	33 (43)	63 (69)	
Second‐line treatment regimen			
Absent	9 (12)	10 (11)	0.437
Oxaliplatin‐based combined	11 (14)	8 (9)	
Irinotecan‐based combined	48 (62)	63 (69)	
Single irinotecan	4 (5)	5 (6)	
Single capecitabine	5 (7)	5 (6)	
Second‐line targeted treatment			
Absent	8 (10)	10 (11)	0.337
Anti‐VEGF	47 (61)	59 (65)	
Anti‐EGFR	17 (22)	13 (14)	
Tumor agnostic targeted drugs	2 (3)	4 (4)	
Immune check‐point inhibitor monotherapy	3 (4)	5 (6)	
Events			
Alive	24 (31)	15 (17)	**0.020**
Death	53 (69)	76 (83)	

*Note:* Data are presented as number (percentage). Categorical variables were compared using the Chi‐squared test or Fisher's exact test, as appropriate. Percentages were calculated based on available data; patients with missing values were excluded from the corresponding analyses. Bonferroni correction was applied for comparisons involving more than two categories. Bold *p*‐values indicate statistical significance (*p* < 0.05).

Abbreviations: BRAF, rapidly accelerated fibrosarcoma; CAPOX, is a combination chemotherapy regimen consists of oxaliplatin and capecitabine that it is typically administered on a 21‐day cycle; ECOG, Eastern Cooperative Oncology Group; EGFR, epidermal growth factor receptor; FOLFIRI, is a combination chemotherapy regimen consists of irinotecan, leucovorin, and fluorouracil (5‐FU) that it is typically administered on a 14‐day cycle; HER2, human epidermal growth factor receptor 2; mFOLFOX6, is a combination chemotherapy regimen consists of oxaliplatin, leucovorin, and fluorouracil (5‐FU) that it is typically administered on a 14‐day cycle; RAS, Kirsten rat sarcoma viral oncogene homolog; VEGF, vascular endothelial growth factor.

^a^ Single agent definition: Only capecitabine or irinotecan.

There were no significant differences in gender (*p* = 0.329), age distribution (*p* = 0.379), smoking status (*p* = 0.406), comorbidity (*p* = 0.724), PPI use before diagnosis (*p* = 0.273), family history of cancer (*p* = 0.532), ECOG performance status (*p* = 0.396), RAS mutation status (*p* = 0.532), microsatellite status (*p* = 0.472), or HER2 amplification/overexpression status (*p* = 0.312).

The distribution of metastatic disease at baseline was also similar between the groups. No significant differences were observed in the presence of liver (*p* = 0.301), lung (*p* = 0.342), bone (*p* = 0.819), brain (*p* = 0.183), distant lymph node (*p* = 0.270) or adrenal gland metastases (*p* = 0.269). Similarly, the number of metastatic sites did not differ significantly between the groups (*p* = 0.096). No statistically significant differences were found between the two groups in terms of chemotherapy backbone regimens (*p* = 0.637), chemotherapy agent subgroup (*p* = 0.548), or targeted therapies (*p* = 0.114). In addition, there were no significant differences in terms of second‐line treatment regimen (*p* = 0.437) and second‐line targeted treatment status (*p* = 0.337).

Right‐sided primary tumors were significantly more frequent in *H. pylori* –positive patients compared with negative patients (43% vs. 17%), whereas left‐sided tumors were more common in the negative group (63% vs. 45%) (*p* < 0.001). BRAF mutation was also more frequently observed in the *H. pylori* –positive group (12% vs. 3%; *p* = 0.044). At the time of analysis, death had occurred in 69% of *H. pylori* –positive patients and 83% of *H. pylori* –negative patients (*p* = 0.020).

Table S1 presents a comparison of patients included and excluded based on *H. pylori* status and treatment suitability. Differences were observed based on first‐line chemotherapy backbone (*p* = 0.037), chemotherapy subgroup (*p* = 0.033), targeted therapy use (*p* = 0.048), ECOG performance status (*p* = 0.033), and primary tumor localization (*p* = 0.045), considering group characteristics. No statistically significant differences were found for other characteristics.

### 3.2 Laboratory Parameters

Baseline laboratory parameters according to *H. pylori* status are presented in Table 2. Median leukocyte count was higher in *H. pylori* –positive patients (*p* = 0.002), as were neutrophil count (*p* = 0.003) and platelet count (*p* = 0.003). CRP levels were also higher in the *H. pylori* –positive group (*p* = 0.033). The NLR ratio was significantly elevated in *H. pylori* –positive patients (*p* = 0.002).

**TABLE 2:** Comparison of laboratory parameters according to *Helicobacter pylori* status.

Variables	*H. pylori* positive	*H. pylori* negative	*p*
Leucocyte count (×10^3^/μL)	8647 (3174–12,167)	7314 (2978–9167)	**0.002**
Neutrophil count (×10^3^/μL)	3974 (2169–7437)	2948 (1979–5137)	**0.003**
Lymphocyte count (×10^3^/μL)	718 (396–1515)	734 (412–1615)	0.699
Platelet count (×10^3^/μL)	394,000 (233,000–745,000)	304,000 (139,000–548,000)	**0.003**
Hemoglobin value (g/dL)	10.2 (7.8–13.7)	10.5 (7.6–13.9)	0.477
Serum LDH level (U/L)	241 (154–312)	247 (159–315)	0.544
Serum uric acid level (mg/dL)	4.86 (1.45–7.14)	4.99 (1.81–7.54)	0.614
Serum albumin level (g/dL)	3.5 (3.1–4.6)	3.9 (3.4–4.6)	0.137
Serum CRP level (mg/L)	32 (11–48)	26 (4–37)	**0.033**
NLR	4.34 (2.26–6.94)	2.74 (1.96–3.42)	**0.002**
NPR	0.018 (0.004–0.034)	0.019 (0.005–0.034)	0.396
MCV	83 (77–91)	85 (76–91)	0.658

*Note:* All laboratory parameters were measured at the time of diagnosis. Continuous variables are presented as median (interquartile range, IQR). Comparisons between groups were performed using the Mann–Whitney *U* test. Bold *p*‐values indicate statistical significance (*p* < 0.05).

Abbreviations: CRP, C‐reactive protein; LDH, lactate dehydrogenase; MCV, mean corpuscular volume; NLR, neutrophil‐to‐lymphocyte ratio; NPR, neutrophil‐to‐platelet ratio.

No significant differences were observed in lymphocyte count (*p* = 0.699), hemoglobin (*p* = 0.477), LDH (*p* = 0.544), uric acid (*p* = 0.614), albumin (*p* = 0.137), neutrophil‐to‐platelet ratio (*p* = 0.396), or MCV (*p* = 0.658).

The overall distribution of baseline laboratory parameters in the entire cohort is summarized in Table S2.

### 3.3 First‐Line Treatment Exposure

First‐line systemic treatment characteristics were similar between the groups (Table S3). Oxaliplatin‐based chemotherapy was the most frequently used backbone in both groups (90% vs. 92%, *p* = 0.548). The distribution of biologic agents was comparable between groups (anti‐VEGF: 62% vs. 51%; anti‐EGFR: 38% vs. 49%; *p* = 0.114), as were maintenance strategies (48% vs. 46%, *p* = 0.216), time from diagnosis to treatment initiation (median 34 [24–52] vs. 35 [24–54] days, *p* = 0.478), treatment cessation (*p* = 0.612), dose reduction (*p* = 0.516), and grade 3–4 toxicity (*p* = 0.395).

Median first‐line PFS was 21 months (95% CI: 10–37) in *H. pylori‐*positive patients and 16 months (95% CI: 4–22) in *H. pylori* ‐negative patients (log‐rank *p* < 0.001).

### 3.4 Survival in the Entire Cohort

When the entire cohort was analyzed irrespective of *H. pylori* status (Table S4), the median OS was 29 months (95% CI: 24–37). Median OS did not differ significantly according to age (< 65 vs. ≥ 65 years: 22 vs. 24 months, *p* = 0.276), smoking status (24 vs. 25 months, *p* = 0.294), comorbidity (22 vs. 24 months, *p* = 0.396), or PPI use (21 vs. 25 months, *p* = 0.341). Similarly, no significant difference was observed according to targeted treatment type (anti‐VEGF vs. anti‐EGFR: 29 vs. 24 months, *p* = 0.412). In contrast, significant differences in survival were observed according to primary tumor localization (*p* < 0.001), with shorter median OS in right‐sided tumors (21 months) compared with left‐sided colon (29 months) and rectal tumors (31 months). The presence of liver metastasis was associated with worse survival (21 vs. 28 months, *p* < 0.001), as was the presence of lung metastasis (22 vs. 29 months, *p* < 0.001).

Patients with RAS‐mutant tumors had shorter median OS compared to those with wild‐type tumors (19 vs. 25 months, *p* < 0.001). Similarly, higher NLR was associated with inferior survival (22 vs. 29 months, *p* < 0.001).

### 3.5 Subgroup Survival Analyses

**FIGURE 2:**
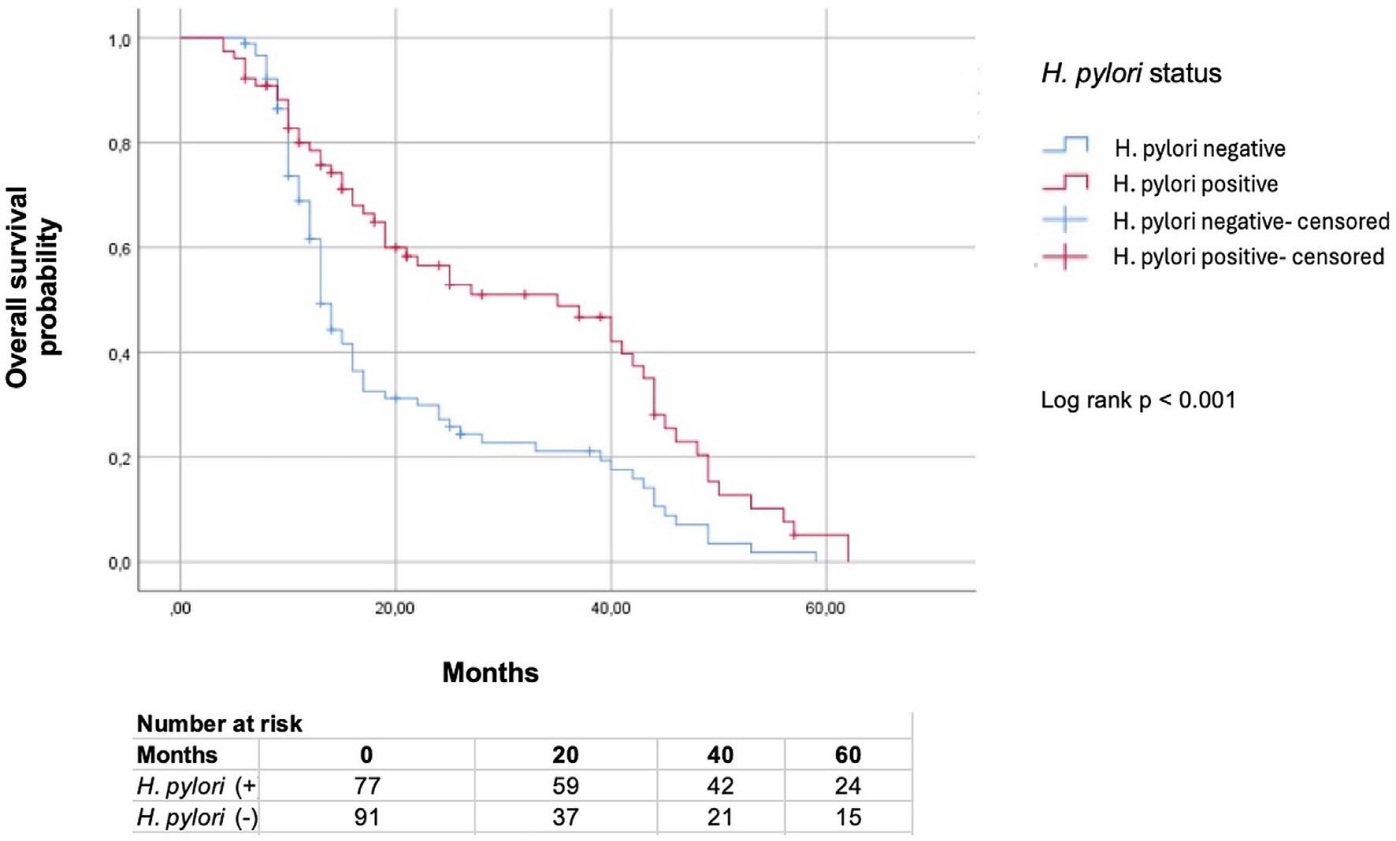
Kaplan–Meier curves for overall survival according to *Helicobacter pylori* status. Kaplan–Meier estimates of overall survival stratified by *Helicobacter pylori* status. Patients with *H. pylori* positivity demonstrated significantly longer overall survival compared with *Helicobacter pylori*–negativepatients (log‐rank *p* < 0.001). Tick marks indicate censored observations. The number of patients at risk at 0, 20, 40, and 60 months is shown below the x‐axis.

Subgroup analyses according to *H. pylori* status are presented in Table S5. Kaplan–Meier survival curves demonstrated differences in OS between groups (Figure 2). Median OS was 33 months (95% CI: 29–37) in the *H. pylori* –positive group and 19 months (95% CI: 16–24) in the negative group (log‐rank *p* < 0.001).

Significant differences in survival were observed in patients with right‐sided tumors (22 vs. 16 months), left‐sided colon tumors (31 vs. 22 months), and rectal tumors (33 vs. 26 months) (*p* < 0.001). Similarly, *H. pylori* –positive patients demonstrated longer OS among those with liver metastasis (26 vs. 17 months, *p* < 0.001), RAS‐mutant tumors (22 vs. 18 months, *p* < 0.001), and higher NLR (24 vs. 17 months, *p* < 0.001). Among patients without lung metastases, *H. pylori* ‐positive patients had longer OS than *H. pylori* ‐negative patients (31 vs. 24 months; *p* = 0.002). In contrast, among patients with lung metastases, survival was similar between the two groups (22 vs. 21 months; *p* = 0.596).

Comparable trends were also observed across treatment‐related subgroups, including targeted therapy (anti‐VEGF: 29 vs. 20 months; anti‐EGFR: 22 vs. 17 months, *p* < 0.001). Although numerical differences favored *H. pylori* –positive patients across most subgroups, some comparisons did not reach statistical significance, including age (*p* = 0.248), sex (*p* = 0.176), smoking status (*p* = 0.209), comorbidity (*p* = 0.287), PPI use (*p* = 0.396), and BRAF mutation status (*p* = 0.112).

### 3.6 Cox Regression Analysis

In the univariable Cox regression analysis (Table 3), right‐sided tumor localization (HR 1.94, 95% CI: 1.673–2.696; *p* < 0.001), RAS mutation (HR 1.91, 95% CI: 1.637–2.737; *p* < 0.001), *H. pylori* negativity (HR 1.96, 95% CI: 1.512–2.794; *p* < 0.001), oxaliplatin‐based chemotherapy regimen (HR 1.48, 95% CI: 1.294–1.966; *p* = 0.003), and targeted treatment exposure (HR 1.76, 95% CI: 1.239–2.437; *p* = 0.004) were associated with differences in OS. BRAF mutation (HR 1.49, 95% CI: 0.712–2.096; *p* = 0.374), age (< 65 vs. ≥ 65 years) (HR 1.09, 95% CI: 0.489–1.967; *p* = 0.344), sex (HR 1.37, 95% CI: 0.564–2.196; *p* = 0.401), and presence of liver metastasis (HR 1.51, 95% CI: 0.642–1.964; *p* = 0.256) were not statistically significant in this analysis.

**TABLE 3:** Cox proportional hazards regression analysis for overall survival.

Features	Univariable analysis				Multivariable analysis			
	HR	Lower 95% CI	Upper 95% CI	*p*	HR	Lower 95% CI	Upper 95% CI	*p*
Age (≥ 65 vs. < 65 years)	1.09	0.489	1.967	0.344	1.04	0.574	1.956	0.306
Gender (female vs. male)	1.37	0.564	2.196	0.401	1.29	0.548	2.096	0.324
Localization (right‐sided vs. left‐sided/rectum)	1.94	1.673	2.696	**< 0.001**	2.11	1.648	2.716	**< 0.001**
Metastatic site (liver vs. no liver metastasis)	1.51	0.642	1.964	0.256	1.44	0.799	1.819	0.248
RAS mutation status (mutant vs. wild‐type)	1.91	1.637	2.737	**< 0.001**	1.94	1.612	2.794	**< 0.001**
BRAF mutation status (mutant vs. wild‐type)	1.49	0.712	2.096	0.374	1.59	0.694	2.144	0.302
*Helicobacter pylori* status (negative vs. positive)	1.96	1.512	2.794	**< 0.001**	1.94	1.569	2.812	**< 0.001**
Targeted treatment (anti‐EGFR vs. anti‐VEGF)	1.76	1.239	2.437	**0.004**	1.37	0.627	2.015	0.109
Chemotherapy regimen (oxaliplatin‐based vs. non‐oxaliplatin‐based)	1.48	1.294	1.966	**0.003**	1.34	0.637	2.245	0.314

*Note:* Variables were included in the multivariable model based on clinical relevance and prior evidence rather than solely on univariable significance. Hazard ratios (HRs) and 95% confidence intervals (CIs) were derived from Cox proportional hazards regression models. Reference categories are shown in parentheses (hazard ratios are presented for the first category relative to the second category). Bold *p*‐values indicate statistical significance (*p* < 0.05).

Abbreviations: CI, confidence interval; EGFR, epidermal growth factor receptor; HR, hazard ratio; RAS, Kirsten rat sarcoma viral oncogene homolog; VEGF, vascular endothelial growth factor.

Variables were included in the multivariable model based on clinical relevance and prior evidence. In the multivariable Cox regression analysis (Table 3), right‐sided tumor localization (HR 2.11, 95% CI: 1.648–2.716; *p* < 0.001), RAS mutation (HR 1.94, 95% CI: 1.612–2.794; *p* < 0.001), and *H. pylori* negativity (HR 1.94, 95% CI: 1.569–2.812; *p* < 0.001) were associated with differences in OS. Other variables, including age, sex, metastatic site, BRAF mutation status, oxaliplatin‐based chemotherapy regimen, and targeted treatment exposure, were not statistically significant in the multivariable model.

### 3.7 Interaction Analyses

In exploratory interaction analyses, no evidence of interaction was observed between *H. pylori* status and age (*p* for interaction = 0.346), RAS mutation status (*p* for interaction = 0.196), PPI use (*p* for interaction = 0.412), or the presence of liver metastasis (*p* for interaction = 0.376). Potential interactions were observed for primary tumor sidedness (*p* for interaction < 0.001) and NLR category (*p* for interaction = 0.002); however, these findings should be regarded as hypothesis‐generating given the limited sample size of several subgroups.

### 3.8 Sensitivity Analyses

Sensitivity analyses using alternative multivariable models are presented in Table 4. Across all model specifications, the association between *H. pylori* status and overall survival remained statistically significant. The hazard ratios for *H. pylori* negativity were consistent across models, including the primary model (HR 1.94, 95% CI: 1.569–2.812; *p* < 0.001), the tumor burden–adjusted model replacing liver metastasis with number of metastatic sites (HR 1.96, 95% CI: 1.533–2.448; *p* < 0.001), the alternative model including NLR as a covariate (HR 1.98, 95% CI: 1.594–2.637; *p* < 0.001), the model restricted to patients receiving oxaliplatin‐based chemotherapy (HR 1.97, 95% CI: 1.591–2.796; *p* < 0.001), and the model restricted to patients receiving targeted drugs in the second‐line setting (HR 1.98, 95% CI: 1.506–2.696; *p* < 0.001) with comparable effect estimates across all alternative model specifications.

**TABLE 4:** Sensitivity analyses of multivariable Cox models evaluating the robustness of the association between *Helicobacter pylori* status and overall survival. Outcome: Overall survival (OS). Effect estimates: Hazard ratio (HR) with 95% confidence interval (CI).

Covariates (reference)	Model 1 (primary model) HR (95% CI)	*p*	Model 2 (tumor burden alternative) HR (95% CI)	*p*	Model 3 (NLR alternative) HR (95% CI)	*p*	Model 4 (oxaliplatin‐based restricted model) HR (95% CI)	*p*	Model 5 (second‐line targeted restricted model) HR (95% CI)	*p*
Age (≥ 65 vs. < 65 years)	1.04 (0.574–1.956)	0.306	1.09 (0.574–1.837)	0.396	1.12 (0.554–1.894)	0.348	1.06 (0.469–2.104)	0.412	1.09 (0.421–2.196)	0.389
Gender (female vs. male)	1.29 (0.548–2.096)	0.324	1.11 (0.509–1.976)	0.322	1.14 (0.496–1.937)	0.296	1.12 (0.438–1.994)	0.216	1.16 (0.491–2.248)	0.348
Localization (right‐sided vs. left‐sided/rectum)	2.11 (1.648–2.716)	**< 0.001**	2.16 (1.609–2.948)	**< 0.001**	2.08 (1.581–2.699)	**< 0.001**	2.14 (1.548–2.637)	**< 0.001**	2.11 (1.435–2.494)	**< 0.001**
Metastatic site (liver vs. no liver metastasis)	1.44 (0.799–1.819)	0.248	—	—	1.48 (0.469–2.294)	0.378	1.43 (0.419–2.193)	0.311	1.42 (0.412–2.114)	0.341
RAS mutation status (mutant vs. wild‐type)	1.94 (1.612–2.794)	**< 0.001**	1.96 (1.337–2.133)	**< 0.001**	1.98 (1.412–2.519)	**< 0.001**	1.92 (1.406–2.337)	**< 0.001**	1.74 (0.418–2.494)	0.262
BRAF mutation status (mutant vs. wild‐type)	1.59 (0.694–2.144)	0.302	1.47 (0.434–2.094)	0.334	1.44 (0.509–2.219)	0.236	1.42 (0.474–2.134)	0.242	1.46 (0.416–2.313)	0.312
*H. pylori* status (negative vs. positive)	1.94 (1.569–2.812)	**< 0.001**	1.96 (1.533–2.448)	**< 0.001**	1.98 (1.594–2.637)	**< 0.001**	1.97 (1.591–2.796)	**< 0.001**	1.98 (1.506–2.696)	**< 0.001**
Targeted treatment in first‐line (anti‐EGFR vs. Anti‐VEGF)	1.37 (0.627–2.015)	0.109	1.41 (0.609–1.989)	0.204	1.39 (0.415–2.396)	0.319	1.42 (0.5 14‐ 2.496)	0.296	1.37 (0.411–2.464)	0.331
Chemotherapy class in first‐line (oxaliplatin‐based vs. non‐oxaliplatin)	1.34 (0.637–2.245)	0.314	1.31 (0.648–1.979)	0.296	1.34 (0.533–1.937)	0.248	—	—	1.33 (0.448–2.014)	0.327
Number of metastatic sites (≥ 3 vs. < 3)	—	—	1.48 (0.548–2.737)	0.327	—	—	—	—	1.91 (1.433–2.637)	**< 0.001**
NLR category (≥ 3 vs. < 3)	—	—	—	—	1.69 (0.537–2.217)	0.236	—	—	—	—
oxaliplatin regimen (CAPOX vs. mFOLFOX6)	—	—	—	—	—	—	1.34 (0.486–2.476)	0.391	—	—
Second‐line targeted treatment (anti‐VGEF vs. anti‐ EGFR vs. ICIs or tumor agnostic drugs)	—	—	—	—	—	—	—	—	1.42 (0.494–2.437)	0.329

*Note:* Definitions: All models were prespecified based on clinical relevance and prior literature. Model 1 (Primary model): Included age, gender, localization, metastatic site, RAS and BRAF mutation status, *H. pylori* status, targeted treatment, and chemotherapy regimen. Model 2 (Alternative tumor burden model): Liver metastasis was replaced by the number of metastatic sites (≥ 3 vs. < 3) to avoid collinearity between metastatic burden variables. Model 3 (Alternative NLR model): NLR was included as an alternative covariate to evaluate whether the association between *H. pylori* status and overall survival was independent of systemic inflammatory status (predefined cut‐off ≥ 3). Model 4 (Oxaliplatin‐based restricted model): analysis restricted to patients receiving oxaliplatin‐based first‐line chemotherapy; chemotherapy regimen was additionally modeled as CAPOX vs. mFOLFOX6. Model 5 (Second‐line restricted model): analysis restricted to patients receiving targeted treatment in second‐line setting; targeted treatment drug was additionally modeled as anti‐VGEF vs. anti‐EGFR vs. ICIs or tumor agnostic drugs. Hazard ratios (HRs) and 95% confidence intervals (CIs) were derived from multivariable Cox proportional hazards regression models. Reference categories are shown in parentheses (Hazard ratios are presented for the first category relative to the second category). Variables not included in a given model are indicated by “—”. Bold *p*‐values indicate statistical significance (*p* < 0.05).

Abbreviations: CAPOX, is a combination chemotherapy regimen that consists of oxaliplatin and capecitabine that is typically administered on a 21‐day cycle; CI, confidence interval; EGFR, epidermal growth factor receptor; HR, hazard ratio; mFOLFOX6, is a combination chemotherapy regimen that consists of oxaliplatin, leucovorin, and fluorouracil (5‐FU) that is typically administered on a 14‐day cycle; RAS, Kirsten rat sarcoma viral oncogene homolog; VEGF, vascular endothelial growth factor.

Because ECOG showed limited variability, with only a very small number of patients having ECOG PS 2, it was not included in the primary multivariable model. In an exploratory ECOG‐adjusted sensitivity analysis, inclusion of ECOG did not materially alter the association between *H. pylori* status and OS; however, the ECOG estimate itself was imprecise, as expected, due to the small number of ECOG PS 2 patients.

## 4 Discussion

The present study suggests that *H. pylori* infection is associated with a distinct clinical phenotype and prolonged OS in patients with mCRC. Although *H. pylori* is well established as a class I carcinogen in gastric malignancies, its role in CRC remains controversial. A meta‐analysis evaluating CRC risk has reported modest but significant associations between *H. pylori* seropositivity and colorectal adenomas, with OR of 1.70 (95% CI: 1.64–1.76, *I* ^2^ = 97%) [19], whereas large population‐based analyses assessing CRC incidence have yielded inconsistent results, with some showing no independent association after multivariable adjustment. Importantly, these studies primarily addressed cancer development rather than outcomes in advanced disease, and data on survival in mCRC are lacking [20, 21, 22, 23, 24]. Against this background, the observed association between histologically confirmed *H. pylori* positivity and longer OS in our cohort suggests that the clinical implications of chronic infection may vary according to disease stage and host–tumor interactions, rather than reflecting a uniform carcinogenic effect across the disease continuum. The observed survival advantage despite enrichment of several adverse prognostic features argues against a simple clinicopathological explanation but also raises the possibility of residual confounding. These findings should be interpreted as exploratory and hypothesis‐generating rather than confirmatory, particularly given the modest sample size and limited subgroup sizes.

One of the most notable observations in the present study was the higher frequency of right‐sided primary tumors among *H. pylori* –positive patients. In large clinical series of mCRC, right‐sided tumors account for approximately 25%–35% of cases and are consistently associated with inferior survival compared with left‐sided disease, with reported median OS differences often exceeding 10–15 months [25, 26]. These tumors are also enriched for BRAF mutations, which are observed in up to 10%–15% of right‐sided metastatic tumors and are linked to particularly poor prognosis [27]. Despite this established adverse biological profile, the association between *H. pylori* status and OS persisted after multivariable adjustment, and a statistically significant interaction between infection status and tumor sidedness was observed. These findings suggest that the relationship between tumor location and survival may differ according to *H. pylori* status within this cohort. However, because interaction analyses were exploratory and several subgroups were relatively small, these observations should be regarded as hypothesis‐generating and require confirmation in larger cohorts before biological inferences can be drawn.

An exception to this pattern was observed among patients with lung metastases. However, given the limited sample size and exploratory nature of subgroup analyses, this finding should be interpreted with caution.

The observed survival difference also prompts consideration of potential immunological mechanisms. Chronic *H. pylori* infection is characterized by sustained activation of both innate and adaptive immune pathways, with documented effects on T‐cell polarization and cytokine signaling [28]. In experimental models and translational studies, persistent antigenic stimulation has been associated with enhanced immune surveillance and modulation of systemic inflammatory responses, indicating that not all forms of chronic inflammation are biologically equivalent [29]. Although direct immune profiling was not available, the coexistence of higher inflammatory indices and longer survival in *H. pylori* –positive patients suggests that systemic inflammatory markers may reflect heterogeneous biological states depending on the underlying context of immune activation. However, this interpretation remains speculative.

Previous studies investigating the relationship between *H. pylori* and colorectal neoplasia have largely relied on serological testing, which reflects past exposure rather than active infection and may result in misclassification rates of up to 20%–30% [6, 20, 21, 30]. In contrast, the present study used synchronous histopathological assessment at the time of metastatic diagnosis, thereby capturing active infection status. Furthermore, while earlier analyses included heterogeneous populations across different disease stages, the current study was restricted to patients with de novo metastatic disease and incorporated comprehensive molecular and clinical variables. In addition, because histological confirmation requires endoscopic sampling, a separate non‐endoscoped comparison group could not be defined using the same exposure definition; therefore, potential selection bias was evaluated through comparison of included and excluded patients. These methodological differences may partly explain the distinct prognostic association observed in our cohort.

The relationship between *H. pylori* status and systemic inflammatory markers further underscores the complexity of inflammation in cancer biology. In large cohorts of mCRC, an NLR cut‐off of 3–5 has consistently been associated with inferior survival, with hazard ratios for mortality ranging from approximately 1.5–2.0 [31, 32]. In the present study, *H. pylori* –positive patients had higher baseline NLR values; however, NLR did not retain independent prognostic significance in the multivariable model, and a significant interaction was observed between NLR category and infection status. These findings suggest that the association between *H. pylori* status and survival is not mediated by NLR. Nevertheless, these observations remain hypothesis‐generating in the absence of direct translational evidence. Therefore, these observations should not be interpreted as evidence of a definitive biological interaction in the absence of external validation and translational correlates.

The predominance of proximal tumors in *H. pylori* –positive patients may also be interpreted within the framework of the gut–microbiome axis [14, 16]. Population‐based microbiome studies have demonstrated substantial spatial variation in bacterial diversity and immune signaling along the colorectum, with higher microbial density and distinct metabolic activity in the right colon. It is therefore conceivable that chronic gastric infection may reflect biological differences in colorectal tumor behavior through systemic immune and inflammatory mechanisms [33, 34]. A key methodological strength of the present study is the synchronous histopathological confirmation of *H. pylori* at diagnosis, which minimizes exposure misclassification and enhances the clinical relevance of the findings.

Taken together, while the available literature on *H. pylori* and colorectal neoplasia is heterogeneous, the present study differs from previous reports in three major aspects: the evaluation of survival rather than cancer risk, the restriction to a clinically homogeneous de novo metastatic population, and the assessment of active infection using histopathology. These features provide a more direct and clinically meaningful insight into the potential prognostic role of chronic microbial exposure in mCRC.

First‐line treatment exposure was well balanced between the groups, including chemotherapy backbone, biologic agent use, treatment initiation time, dose modifications, and toxicity profiles. Second‐line treatment characteristics were also incorporated into the analyses to further address potential treatment‐related confounding. Across multiple sensitivity analyses, including alternative model specifications, restriction to oxaliplatin‐based regimens, and adjustment for metastatic burden and NLR, the association between *H. pylori* status and OS remained consistent.

Recent large population‐based studies have suggested that *H. pylori* infection may be associated with increased CRC incidence and mortality. However, these studies primarily evaluated cancer risk or population‐level outcomes rather than survival in patients with established metastatic disease [35, 36, 37, 38, 39, 40]. In contrast, the present study specifically addresses OS in a clinically homogeneous de novo mCRC cohort with histologically confirmed active infection. Because *H. pylori* status was determined based on histopathological assessment obtained during clinically indicated endoscopy, the findings should be interpreted within the context of potential selection mechanisms inherent to real‐world clinical practice.

Several limitations should be acknowledged. First, the retrospective and single‐center design introduces the potential for selection bias and limits the generalizability of the findings. Because *H. pylori* status was defined based on histopathological assessment obtained during endoscopy, a comparable control group of patients without endoscopic evaluation could not be established using the same exposure definition. Therefore, the study population represents a selected subgroup of patients who underwent synchronous endoscopic assessment at diagnosis. Because the study included patients who underwent endoscopy based on clinical indications, the cohort may not fully represent an unselected mCRC population. Although baseline characteristics were broadly comparable, differences in treatment‐related variables between included and excluded patients suggest that selection related to endoscopy availability may have influenced cohort composition and potentially the observed outcomes. Importantly, comparison of included and excluded patients documented differences in first‐line chemotherapy backbone, chemotherapy subgroup, and targeted treatment exposure, suggesting that the analyzed cohort differed clinically from the broader institutional de novo metastatic CRC population. Therefore, Table S1 should be interpreted as descriptive of cohort composition rather than reassuring against selection bias. Residual selection mechanisms related to endoscopy indication, treatment eligibility, performance status, frailty, or nutritional assessment may have influenced the observed associations.

Given the observational design and the magnitude of the observed effect, residual and unmeasured confounding may have influenced the association between *H. pylori* status and OS. Although adjustments were made for clinically relevant variables, including treatment‐related factors, it is possible that unrecorded factors such as detailed treatment selection processes, post‐progression therapies, tumor burden characteristics, and patient‐related factors (e.g., frailty or nutritional status) contributed to the findings. Therefore, the observed association should be interpreted cautiously and cannot be considered causal. An additional observation supporting the consistency of the findings is the association between *H. pylori* status and first‐line PFS. H. *pylori‐*positive patients demonstrated not only longer OS, but also longer PFS compared with *H. pylori* ‐negative patients. Although PFS assessment was retrospective and based on routine clinical practice rather than formal RECIST review, the concordance between OS and PFS findings argues against the possibility that the observed survival difference was solely driven by post‐progression treatment factors. Nevertheless, these results should be interpreted cautiously and require prospective validation.

Second, the relatively small number of patients in certain molecular subgroups, particularly those with BRAF‐mutant and MSI‐high tumors, limited the statistical power of subgroup analyses and precluded more detailed interaction modeling. Third, information regarding *H. pylori* eradication prior to the diagnosis of metastatic disease was not available. However, none of the patients received eradication therapy after the diagnosis, as the clinical management was focused on systemic anticancer treatment. In routine clinical practice, eradication therapy is rarely prioritized in the setting of newly diagnosed metastatic disease, where systemic treatment represents the primary therapeutic focus. In addition, exposure to short‐term antibiotic or bismuth‐containing regimens could not be systematically captured from retrospective records, which may have influenced *H. pylori* detection status. Furthermore, *H. pylori* status was assessed only at baseline, and longitudinal information regarding persistence of infection or eradication following diagnosis was not available. Although ECOG performance status was available, the distribution was highly skewed, with the vast majority of patients having ECOG PS 0–1 and only a small number having ECOG PS 2. Therefore, ECOG was not included in the primary multivariable model because reliable estimation of its prognostic effect was limited. Although first‐line treatment strategies and exposure parameters were comparable between the groups, the possibility of residual confounding related to unmeasured treatment variables cannot be entirely excluded. Because *H. pylori* status was determined at the time of diagnosis through synchronous endoscopic assessment, the risk of immortal time bias was minimized. Finally, the absence of translational correlates, including immune profiling, tumor‐infiltrating lymphocyte assessment, and microbiome analyses, limits the biological interpretation of the observed associations.

Future prospective studies integrating immune profiling, tumor‐infiltrating lymphocyte quantification, and microbiome analyses are needed to clarify the biological mechanisms underlying these observations. Such investigations may also help to determine whether chronic microbial exposure interacts with contemporary systemic therapies, including immune checkpoint inhibitors, particularly in right‐sided or BRAF‐mutant CRC.

From a clinical perspective, the present findings suggest that assessment of *H. pylori* status at the time of diagnosis may provide additional prognostic information in patients with de novo mCRC, particularly when interpreted together with established molecular and inflammatory markers. Given the retrospective and observational design of this study, a causal relationship between *H. pylori* infection and survival outcomes cannot be inferred, and the observed association should be interpreted as hypothesis‐generating. In summary, *H. pylori* positivity was associated with a distinct clinicopathological profile and prolonged overall survival in this real‐world mCRC cohort, raising the possibility of an interaction between chronic microbial exposure, host immunity, and tumor biology that warrants further prospective and translational investigation.

## 5 Conclusion

In conclusion, the present study suggests that *H. pylori* positivity is associated with longer OS in patients with mCRC and may have prognostic relevance in this setting. The observed co‐occurrence of *H. pylori* infection with right‐sided tumors and higher systemic inflammatory markers points to a complex interaction between chronic microbial exposure, host immunity, and tumor biology. Interestingly, the prognostic effect estimate associated with *H. pylori* status was numerically similar to that observed for established molecular prognostic factors such as RAS mutation. Given the limited sample size and retrospective design, this observation should not be interpreted as evidence that *H. pylori* itself exerts a prognostic influence equivalent to major tumor‐specific molecular drivers. Rather, *H. pylori* status may reflect broader host‐related biological processes that are incompletely captured by conventional clinicopathological variables. Notably, *H. pylori* ‐positive patients demonstrated a survival advantage despite a higher prevalence of several traditionally adverse prognostic features, including right‐sided primary tumors, BRAF mutations, and elevated inflammatory markers. While this observation argues against a simple clinicopathological explanation, residual confounding and selection‐related factors cannot be excluded. Although causality cannot be inferred from this observational analysis, these findings suggest that *H. pylori* status may provide additional clinical and biological insight in mCRC and support further investigation in prospective and translational studies.

## Author Contributions

O.T. conceived the study, collected data, performed data analysis, and edited the manuscript. S.K. and A.A. collected data and drafted the manuscript. All authors reviewed the manuscript.

## Funding

The authors have nothing to report.

## Ethics Statement

Medical and Health Sciences Ethics Committee of Mugla Sitki Kocman University (Ethics Committee Approval Number: 250115/82, Date: January 16, 2026). All procedures were conducted following the ethical principles outlined in the Declaration of Helsinki.

## Consent

Given the retrospective nature of the study, informed consent was waived by the Medical and Health Sciences Ethics Committee of Mugla Sitki Kocman University.

## Conflicts of Interest

The authors declare no conflicts of interest.

## Supporting information


**Table S1:** Baseline clinicopathological characteristics of included and excluded patients stratified by *Helicobacter pylori* status availability and treatment eligibility.
**Table S2:** Distribution of baseline laboratory parameters in the study population (*n* = 168).
**Table S3:** First‐line treatment characteristics and outcomes according to *Helicobacter pylori* status.
**Table S4:** Overall survival according to clinicopathological characteristics in the study cohort.
**Table S5:** Subgroup analysis of overall survival according to *Helicobacter pylori* status in the study cohort.

## Data Availability

Additionally, as this study relies on institutional patient records, the data are not publicly available due to confidentiality agreements. Researchers interested in accessing the dataset may request it from the corresponding author, subject to institutional approval.
