# Supplementary material for: Active Helicobacter pylori Infection and Survival Outcomes in De Novo Metastatic Colorectal Cancer: A Retrospective Cohort Study
Source: Helicobacter. 2026 Jul 6;31(4):e70150. doi: 10.1111/hel.70150 (PMC13338582; doi:10.1111/hel.70150)
Supplement: Supplementary file 1 — Table S1: Baseline clinicopathological characteristics of included and excluded patients stratified by Helicobacter pylori status availability and treatment eligibility. Table S2: Distribution of baseline laboratory parameters in the study population (n = 168). Table S3: First‐line treatment characteristics and outcomes according to Helicobacter pylori status. Table S4: Overall survival according to clinicopathological characteristics in the study cohort. Table S5: Subgroup analysis of overall survival according to Helicobacter pylori status in the study cohort. [file HEL-31-e70150-s001.docx]

**Supplementary Table S1.** Baseline clinicopathological characteristics of included and excluded patients stratified by *Helicobacter pylori* status availability and treatment eligibility

| **Characteristics** | **Study Patients**  **(H. pylori status proven by biopsy)**  **(n=168)** | | **Excluded Patients**  **(H. pylori status not proven by biopsy)**  **(n=50)** | | **Excluded Patients**  **(H. pylori status proven by biopsy)**  **(n=30)** | | **P value** |
| --- | --- | --- | --- | --- | --- | --- | --- |
|  | **n** | **%** | **n** | **%** | **n** | **%** |  |
| Gender  Female  Male | 58  110 | 34  66 | 20  30 | 40  60 | 10  20 | 33  67 | 0.437 |
| Age  <65  65 and over | 75  93 | 45  55 | 24  26 | 48  52 | 11  19 | 37  63 | 0.396 |
| Smoking habits  Never/ Ex-smoker  Active smoker | 124  44 | 74  26 | 33  17 | 66  34 | 21  9 | 70  30 | 0.333 |
| Comorbidity  Present  Absent  Unknown | 93  67  8 | 55  40  5 | 22  24  4 | 44  48  8 | 19  9  2 | 63  31  7 | 0.348 |
| Proton pump inhibitors use  Present  Absent  Unknown | 66  82  20 | 39  49  12 | 19  21  20 | 38  42  40 | 14  14  2 | 47  47  7 | 0.069 |
| Cancer history in family  Present  Absent  Unknown | 25  57  86 | 15  34  51 | 11  19  20 | 22  38  40 | 9  12  9 | 30  40  30 | 0.266 |
| Performance status (ECOG)  0  1  2 | 20  143  5 | 12  85  3 | 1  41  8 | 2  82  16 | 5  24  1 | 17  80  3 | **0.033** |
| Primary tumor localization  Right colon  Left colon  Rectum | 49  92  27 | 29  55  16 | 26  15  9 | 52  30  18 | 9  13  8 | 30  43  27 | 0.045 |
| Number of metastatic sites  1  2  3 and over | 33  57  78 | 20  34  46 | 9  21  20 | 18  42  40 | 12  8  10 | 40  27  33 | 0.054 |
| Metastatic site at time of diagnosis  Liver  Lung  Bone  Brain  Distant lymph node  Adrenal gland | 105  65  22  7  61  37 | 63  39  13  4  36  22 | 37  34  33  2  29  22 | 74  68  66  8  58  44 | 21  19  9  2  11  14 | 70  63  31  6  37  47 | 0.337 |
| Chemotherapy backbone (first-line)  mFOLFOX6  CAPOX  FOLFIRI  Other (single-agent*)  Absent | 84  69  6  9  0 | 50  41  4  5  0 | 33  9  2  5  1 | 66  18  8  10  2 | 6  8  2  6  8 | 20  27  7  20  27 | **0.037** |
| Chemotherapy subgroup (first-line)  Oxaliplatin-based  Irinotecan-based  Capecitabine  Absent | 153  11  4  0 | 91  6  3  0 | 42  3  4  1 | 84  6  8  2 | 14  3  5  8 | 47  10  17  27 | **0.033** |
| Targeted treatment  Anti- VEGF  Anti- EGFR  Absent | 94  74  0 | 56  44  0 | 29  17  4 | 58  34  8 | 11  13  6 | 37  43  20 | **0.048** |
| RAS mutation status  Mutant  Wild  Unknown | 54  114  0 | 32  68  0 | 19  29  2 | 38  58  4 | 14  16  0 | 47  53  0 | 0.196 |
| BRAF mutation status  Mutant  Wild  Unknown | 12  156  0 | 7  93  0 | 6  37  7 | 12  74  14 | 4  22  4 | 13  73  13 | 0.064 |
| Microsatellite status  Stable  Instable  Unknown | 128  10  30 | 76  6  18 | 35  6  9 | 70  12  18 | 19  3  8 | 63  10  27 | 0.054 |
| HER2 amplification/overexpression  Present  Absent  Unknown | 2  70  96 | 1  42  57 | 0  37  13 | 0  74  26 | 1  21  8 | 3  70  27 | 0.051 |

Abbreviations: ECOG: Eastern Cooperative Oncology Group; mFOLFOX6: is a combination chemotherapy regimen consists of oxaliplatin, leucovorin, and fluorouracil (5-FU) that it is typically administered on a 14-day cycle; CAPOX: is a combination chemotherapy regimen consists of oxaliplatin and capecitabine that it is typically administered on a 21-day cycle; FOLFIRI: is a combination chemotherapy regimen consists of irinotecan, leucovorin, and fluorouracil (5-FU) that it is typically administered on a 14-day cycle; VEGF: vascular endothelial growth factor; EGFR: epidermal growth factor receptor; RAS: Kirsten rat sarcoma viral oncogene homolog; BRAF: rapidly accelerated fibrosarcoma; HER2: human epidermal growth factor receptor 2.

Baseline characteristics were compared between included and excluded patients to evaluate potential selection bias. Data are presented as number (percentage). Percentages were calculated based on the total number of patients in each group. Excluded patients comprised two groups: (1) patients in whom H. pylori status could not be assessed due to the absence of synchronous gastroscopy at diagnosis, most commonly because of emergency surgical presentation; and (2) patients with known H. pylori status who were excluded from the final analysis due to predefined criteria, including receipt of local ablative or surgical treatment for metastatic disease at baseline, lack of first-line systemic therapy, missing data for key variables, or clinical ineligibility for systemic treatment (e.g., poor performance status, significant comorbidities, or advanced age).

Patients may have more than one metastatic site at the time of diagnosis. P values were calculated using the chi-square or Fisher’s exact test, as appropriate.

**Supplementary Table 2.** Distribution of baseline laboratory parameters in the study population (n=168)

| **Variables** | **Median (interquartile range)** |
| --- | --- |
| **Leucocyte count** (×10³/µL) | 7948 (2978-12167) |
| **Neutrophil count** (×10³/µL) | 3478 (1979-7437) |
| **Lymphocyte count** (×10³/µL) | 748 (396-1615) |
| **Platelet count** (×10³/µL) | 337 000 (139 000- 745 000) |
| **Hemoglobin value (**g/dL) | 10.4 (7.6-13.9) |
| **Serum LDH level (**U/L) | 245 (154-315) |
| **Serum uric acid level (**mg/dL) | 4.96 (1.45-7.54) |
| **Serum albumin level (**g/dL) | 3.7 (3.1-4.6) |
| **Serum CRP level (**mg/L) | 33 (4-48) |
| **NLR** | 2.84 (1.96-5.2) |
| **NPR** | 0.019 (0.004-0.034) |
| **MCV** | 84 (76-91) |

Abbreviations: LDH, lactate dehydrogenase; CRP, C-reactive protein; NLR, neutrophil-to-lymphocyte ratio; NPR, neutrophil-to-platelet ratio; MCV, mean corpuscular volume.

Continuous variables are presented as median (interquartile range, IQR).

All laboratory parameters were measured at the time of diagnosis. These variables were used for descriptive purposes and to derive inflammatory indices such as NLR.

**Supplementary Table S3.** First-line treatment characteristics and outcomes according to *Helicobacter pylori* status

| **Variable** | ***Helicobacter pylori* positive (n=77)** | ***Helicobacter pylori* negative (n=91)** | **p value** |
| --- | --- | --- | --- |
| **Maintenance strategy; n (%)**  Yes  No | 37 (48)  40 (52) | 42 (46)  49 (54) | 0.216 |
| **Time from diagnosis to first-line treatment start, days; median (range)** | 34 (24-52) | 35 (24-54) | 0.478 |
| **Treatment cessation; n (%)**  Yes  No | 6 (8)  71 (92) | 9 (10)  82 (90) | 0.612 |
| **>10% dose reduction at the 1^st^ line setting; n (%)**  Yes  No | 11 (22)  60 (78) | 18 (20)  73 (80) | 0.516 |
| **Grade 3-4 toxicity; n (%)**  Yes  No | 4 (5)  73 (95) | 7 (8)  84 (92) | 0.395 |
| **PFS1 (Months); median (95% CI)** | 21 (10- 37) | 16 (4- 22) | **<0.001** |

Data are presented as n (%) unless otherwise specified.

Continuous variables are expressed as median (range), except PFS, which is presented as median (95% CI) estimated by the Kaplan–Meier method.

PFS1 was defined as the time from initiation of first-line systemic therapy to disease progression or death from any cause.

Treatment cessation refers to discontinuation of first-line therapy for any reason.

P values were calculated using the chi-square or Fisher’s exact test for categorical variables and the Mann–Whitney U test for continuous variables.

**Supplementary Table S4.** Overall survival according to clinicopathological characteristics in the study cohort

| **Subgroups** | **All Patients**  Median (95% CI), months) | **Log-rank p** |
| --- | --- | --- |
| **Overall cohort** | 29 (24 -37) |  |
| **Age (years)**  <65  ≥65 | 22 (17- 31)  24 (19- 33) | 0.276 |
| **Smoking history**  Present  Absent | 24 (19- 31)  25 (21- 34) | 0.294 |
| **Comorbidity**  Present  Absent | 22 (18- 29)  24 (21- 32) | 0.396 |
| **PPI use before diagnosis**  Present  Absent | 21 (17-30)  25 (19- 32) | 0.341 |
| **Primary tumor localization**  Right colon  Left colon  Rectum | 21 (16- 29)  29 (21- 33)  31 (24- 36) | **<0.001** |
| **Targeted treatment**  Anti- VEGF  Anti- EGFR | 29 (22- 34)  24 (20- 32) | 0.412 |
| **Liver metastasis**  Present  Absent | 21 (16- 29)  28 (24- 37) | **<0.001** |
| **Lung metastasis**  Present  Absent | 22 (16- 28)  29 (21- 35) | **<0.001** |
| **RAS mutation status**  Mutant  Wild | 19 (14- 26)  25 (19- 34) | **<0.001** |
| **NLR**  Higher  Lower | 22 (16- 31)  29 (22- 36) | **<0.001** |

Abbreviations: CI, confidence interval; PPI, proton pump inhibitor; VEGF, vascular endothelial growth factor; EGFR, epidermal growth factor receptor; RAS, Kirsten rat sarcoma viral oncogene homolog; NLR, neutrophil-to-lymphocyte ratio.

Overall survival is presented as median (95% CI) in months. NLR was categorized using a predefined cut-off value of ≥3.

P values were calculated using the log-rank test.

**Supplementary Table S5.** Subgroup analysis of overall survival according to *Helicobacter pylori* status in the study cohort

| **Subgroups** | **H. pylori positive**  Median (95% CI), months | **H. pylori negative**  Median (95% CI), months | **Log-rank p** |
| --- | --- | --- | --- |
| **Overall cohort** | 33 (29- 37) | 19 (16- 24) | **<0.001** |
| **Age (years)**  <65  ≥65 | 24 (21-29)  29 (26- 35) | 22 (19-28)  25 (22- 31) | 0.248 |
| **Gender**  Female  Male | 24 (21-30)  28 (25-36) | 21 (18-29)  24 (19-30) | 0.176 |
| **Smoking history**  Present  Absent | 22 (18- 29)  24 (19-30) | 21 (17- 29)  22 (18- 29) | 0.209 |
| **Comorbidity**  Present  Absent | 21 (17- 30)  25 (19-32) | 20 (16- 28)  22 (18- 30) | 0.287 |
| **PPI use before diagnosis**  Present  Absent | 22 (18- 30)  24 (19- 31) | 19 (15-22)  22 (17-26) | 0.396 |
| **Primary tumor localization**  Right colon  Left colon  Rectum | 22 (18 -29)  31 (24- 36)  33 (28- 37) | 16 (11- 21)  22 (19- 29)  26 (21- 31) | **<0.001** |
| **Chemotherapy backbone (first-line)**  mFOLFOX  CAPOX  FOLFIRI  Single agent* | 26 (21-32)  24 (19-31)  22 (17-29)  21 (17-28) | 21 (17-26)  17 (12-21)  16 (10-20)  15 (10-19) | 0.055 |
| **Targeted treatment**  Anti- VEGF  Anti- EGFR | 29 (22- 37)  22 (19- 30) | 20 (17- 27)  17 (11- 21) | **<0.001** |
| **Liver metastasis**  Present  Absent | 26 (21- 35)  31 (26- 38) | 17 (12- 25)  24 (19- 30) | **<0.001** |
| **Lung metastasis**  Absent  Present | 31 (24- 37)  22 (18- 30) | 24 (19- 33)  21 (17- 31) | **0.002** |
| **RAS mutation status**  Mutant  Wild | 22 (17- 31)  30 (24- 38) | 18 (11- 22)  22 (17- 30) | **<0.001** |
| **BRAF mutation**  Mutant  Wild | 26 (19-33)  31 (22-37) | 20 (17-30)  25 (19-32) | 0.112 |
| **NLR**  Higher  Lower | 24 (18- 31)  30 (24- 38) | 17 (11- 22)  22 (16- 30) | **<0.001** |

Abbreviations: PPI, proton pump inhibitor; VEGF, vascular endothelial growth factor; EGFR, epidermal growth factor receptor; RAS, Kirsten rat sarcoma viral oncogene homolog; NLR, neutrophil-to-lymphocyte ratio.

Overall survival was estimated using the Kaplan–Meier method and compared using the log-rank test.

Survival estimates are presented as median (95% CI) in months.

P values for variables with more than two categories were calculated using the log-rank test across all groups.

Based on prior studies in metastatic colorectal cancer and large meta-analyses, an NLR value of ≥3 was used as the cut-off.
